# Rotational Stability in a Feline Sacroiliac Luxation Model: Biomechanical Comparison of Cannulated Compression Headless Screws and Cortical Screws Applied in Positional or Lag Fashion

**DOI:** 10.3390/ani16101564

**Published:** 2026-05-21

**Authors:** Jana Klement, Josef Wieser, Christoph Thorwächter, Yury Zablotski, Nina Dorothee Lorenz, Susanne Lauer, Matthias Kornmayer

**Affiliations:** 1 Veterinary Practice Dr. Zauscher, 85235 Odelzhausen, Germany; janaklement@gmail.com; 2 Department of Orthopedics and Trauma Surgery, Musculoskeletal University Center Munich (MUM), University Hospital, LMU Munich, 81377 Munich, Germany; josef.wieser@med.uni-muenchen.de (J.W.); christoph.thorwaechter@med.uni-muenchen.de (C.T.); 3 LMU Small Animal Clinic, Centre for Clinical Veterinary Medicine, Ludwig-Maximilians-Universität, 80539 Munich, Germany; y.zablotski@med.vetmed.uni-muenchen.de (Y.Z.); s.lauer@lmu.de (S.L.); 4 Northern Rivers Veterinary Specialists, Bangalow, NSW 2479, Australia

**Keywords:** sacroiliac luxation, headless compression screw, positional screw, lag screw, biomechanical study, rotational stability, feline, cat

## Abstract

The purpose of this study was to compare the rotational stability of cannulated compression headless screws (CCHSs) with cortical positional and lag screws in a simulated cadaveric feline sacroiliac luxation (SIL) model. Thirty-six cadavers of domestic short-haired cats were assigned to three groups. A unilateral SIL model was stabilized using either a single 2.5 mm CCHS, a 2.4 mm cortical positional screw (PS) or lag screw (LS), each engaging 60% of the sacral width. The pelves were mounted in a mechanical testing machine and loaded through the acetabulum to simulate ground reaction forces. Constant displacement was applied at 0.5 mm/s until maximal failure occurred. Yield load, ultimate load, stiffness, and failure modes were compared among the three groups. At the yield load, a statistical trend was observed among the groups, and pairwise comparison showed a significant difference between CCHS and PS. However, no significant differences were identified among the groups for yield load, ultimate load, or stiffness. All specimens failed due to screw loosening, and failure modes differed between CCHSs and cortical screws. Overall, CCHSs and cortical screws placed in a positional or lag fashion showed comparable rotational stability under the single load-to-failure conditions used in this feline SIL model. These findings question the biomechanical advantage of compression screws regarding rotational strength in unilateral feline SIL.

## Simple Summary

Sacroiliac luxation (SIL) is a common injury in cats, and surgical treatment using compression screws is currently the recommended fixation technique. In recent years, minimally invasive procedures using cannulated headless compression screws have increasingly become standard. These screws are partially threaded and are designed to improve stability and promote healing. This experimental study evaluated whether cannulated compression headless screws (CCHSs) provide greater stability than traditionally used fully threaded screws in the treatment of SIL in cats. In addition, conventional screws applied either with or without compression were assessed. Interestingly, CCHSs showed stability comparable to that of conventional screws. Conventional screws applied with compression also demonstrated similar stability to those applied without compression. In addition, non-compressing, fully threaded conventional screws may provide greater stability; however, this finding requires further investigation. The results suggest that compression may not be essential and that fully threaded screws could be a suitable option for minimally invasive treatment of feline SIL, potentially challenging current recommendations. Nevertheless, further biomechanical studies are needed to confirm these observations.

## 1. Introduction

Sacroiliac luxations (SILs) are common in cats, accounting for 59–93% of all pelvic fractures, and are usually unilateral [1,2]. Excellent outcomes have been reported in a limited number of conservatively treated cases, supporting current recommendations for non-surgical management [3]. However, conservative management may lead to long-term degenerative joint disease in the sacroiliac and lumbosacral joints [4,5,6], and its effect on functional outcome remains unclear [3]. Because SIL disrupts the pelvic limb weight-bearing axis [4,7], surgical stabilization may reduce pain, accelerate recovery, and preserve normal pelvic canal diameter [8,9,10,11].

Lag screw fixation spanning 60% of the sacral width is currently the preferred surgical method for unilateral SILs [12,13]. Minimally invasive techniques have become standard owing to technical advances and improved access to intraoperative imaging [12]. Fluoroscopic-guided closed reduction with percutaneous insertion of cannulated screws over a guide wire improves implant positioning, reduces screw loosening, and yields good to excellent functional outcomes in cats [14,15,16]. In 31 SILs in 25 cats, fixation with a single self-drilling, self-tapping, partially threaded 2.4 mm or 3.0 mm headless compression screw (HCS) showed no loosening on short-term radiographic follow-up in unilateral cases [15,16]. Headless compression screws are designed to enhance interfragmentary compression, thereby improving fixation stability and healing [17,18,19,20,21]. Biomechanical studies in dogs have evaluated screw type, number, size, length and thread direction for SIL stabilization [22,23,24,25]. In cats, only one study has assessed biomechanics, comparing fixation methods for unilateral SIL in 3D-printed models [26]. It was found that double J-shaped Kirschner wires provided greater rotational stability than single lag screws with transiliac pinning and double parallel Kirschner wires [26]. However, no studies have evaluated the biomechanical properties of HCSs or single conventional screws for unilateral SILs in cats.

The objective of this study was to evaluate the rotational stability of a single cannulated compression headless screw (CCHS) in a simulated SIL model and compare it with cortical positional and lag screws.

Based on screw design [17,27], we hypothesized that a single CCHS engaging 60% of the sacral width was expected to provide greater stability than cortical screws applied in positional or lag fashion, and that cortical lag screws may be stronger than positional screws.

## 2. Materials and Methods

### 2.1. Specimens

Forty-six feline cadavers were used. All cats had died or were euthanized for reasons unrelated to this study. Age was unknown in 12 cats. Inclusion criteria were domestic shorthair breed, skeletal maturity and normal pelvic anatomy. Orthogonal pelvic radiographs (Luminos dRF Max, Siemens Healthcare GmbH, 91301 Forchheim, Germany) confirmed skeletal maturity and excluded orthopedic disease of the coxofemoral and sacroiliac joints. All specimens met these inclusion criteria. Pelves were isolated at L7-S1 and S3-Cd1, and the coxofemoral joints were disarticulated. Soft tissues were removed except for those associated with the sacroiliac joint. Ten specimens were used for preliminary testing of cortical screw insertion torque and randomly assigned to positional or lag screw groups (*n* = 5 each; Microsoft Excel, Microsoft Corporation, Redmond, WA, USA). The remaining 36 pelves were randomly allocated to three groups (*n* = 12 each): cannulated compression headless screw (CCHS), cortical positional screw (PS), and cortical lag screw (LS). Specimens were wrapped in saline-soaked gauze and stored at −20°.

### 2.2. Radiographic Determination of Screw Length

Implant length was determined on calibrated ventrodorsal radiographs by one board-certified surgeon (MK) to span approximately 60% of the sacral width, as recommended [5]. Left ilium width, sacroiliac joint space, and sacral width were measured at the cranio-caudal midpoint of the sacral body [13].

### 2.3. Implants

For feline SIL stabilization, CCHSs are available in sizes of 2.0 mm, 2.5 mm, and 3.0 mm [27]. In two feline clinical studies, HCSs of 2.4 mm and 3.0 mm were used [15,16]. For comparison of rotational stability, implant sizes with only minimal differences were selected.

**Figure 1:**
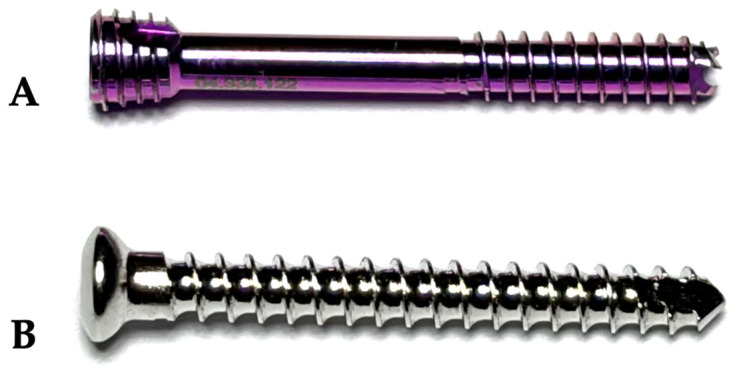
Photograph illustrating the implants tested for their rotational strength in a simulated feline unilateral sacroiliac luxation model, stabilized with either a 2.5 mm partially threaded cannulated compression headless screw (CCHS, DePuy Synthes) (**A**) or a 2.4 mm fully threaded cortical screw (DePuy Synthes) applied in a lag or positional fashion (**B**).

Therefore, the CCHS group received 2.5 mm short threaded, self-drilling, self-tapping, titanium alloy cannulated compression headless screws (DePuy Synthes, Johnson&Johnson, West Chester, PA, USA) (Figure 1). The PS and LS groups received 2.4 mm fully threaded, self-tapping stainless steel cortical screws (DePuy Synthes) (Figure 1).

### 2.4. Preimplantation Preparation

Specimens were thawed at room temperature for 24 h. All procedures were performed by one board-certified surgeon (MK). A 1.0 mm Kirschner wire (K-wire; Mede Technik GmbH, Emmingen-Liptingen, Germany) was inserted from left to right through the centre of the sacral body, perpendicular to the dorsal spinous process and parallel to the cranial endplate of S1 [5]. Placement was confirmed fluoroscopically in orthogonal views (SIREMOBIL Compact L, Siemens AG, 91301 Forchheim, Germany). K-wire depth was measured (direct measuring device for CCHS 2.5/3.0 mm, DePuy Synthes) and matched to the predetermined screw length. For CCHS placement, the K-wire tract was overdrilled with a 2.0 mm cannulated drill bit (DePuy Synthes). For cortical screws, the tract was sequentially enlarged (1.2, 1.4, and 1.5 mm K-wires) and drilled to 1.8 mm. Localization and depth of enlarging K-wires and the drill bits were controlled using marked instruments and verified fluoroscopically in orthogonal views. For lag screws, a 2.4 mm gliding hole was drilled through the ilium.

The sacroiliac joint capsule was transected using a scalpel blade, and the left pubis and ischium were osteotomized at their ipsilateral levels using an oscillating saw (Colibri II, DePuy Synthes). The right hemipelvis was discarded. Left hemipelves with sacra were wrapped in saline-soaked gauze and refrozen at −20 °C.

### 2.5. Evaluation of Screw Insertion Torque

A preliminary test determined mean stripping torque for cortical screws (length 22–24 mm). Screw insertion was performed using a calibrated electronic torque screwdriver (ANPUDS^®^, Hefei Zhidi Network Technology Co., Ltd, Hefei, China). Mean stripping torque was 0.25 Nm for PS and 0.32 Nm for the LS. Target insertion torque was set at 80% of these values [28]: 0.20 Nm (PS) and 0.26 Nm (LS).

### 2.6. Implantation Technique and Mechanical Testing

Mechanical testing followed a previously described canine model simulating hindlimb ground reaction forces [22].

Specimens were thawed for 24 h. Approximately one third of the right sacrum was embedded in a metal fixture using fast-curing resin (SikaBiresin^®^, Sika AG, Baar, Switzerland). Specimens were positioned at a 75° sagittal plane angle to simulate a feline standing posture [29].

Following hardening, a pressure-mapping sensor with a central hole was placed in the sacroiliac joint to measure compressive forces [30]. The ilium was reduced, and screws were inserted through the ilium and sensor into the sacrum. Cortical screws were tightened to predefined torque values; CCHSs were inserted until full thread engagement.

The moment arm was measured (cm) with a digital caliper to calculate rotational forces [22]. The construct was mounted in a testing machine (Instron Worldwide Headquarters, 825 University Ave, Norwood, MA 02062-2643, USA Main). A hemispherical screw simulating the femoral head was embedded in a fixture pot and allowed free movement to ensure purely vertical loading. The craniocaudal translation used to replicate physiologic conditions represented a minor modification of the protocol described in a similar canine study [22].

Rotational force was applied by displacing the hemipelvis downward at 0.5 mm/s until failure. Load–displacement curves (N/mm) were recorded. Yield load (N) was defined as the first decrease in load (deviation from linearity). The ultimate load (maximum failure load) was defined at a rotational displacement of 5°. Human biomechanical data report a mean physiological range of motion of approximately 2.5° [31,32], with one study describing sacroiliac joint motion ranging from 0.8° to 3.9° and linear translation peaking at 1.6 mm [31]. Comparable data for the feline sacroiliac joint are currently lacking. Pre-tests indicated that at a displacement of 5° exceeds the elastic limit of the sacroiliac joint. Under excessive rotational loading, the incongruity of the C-shaped articular surface forces the ilium out of the sacral socket, and the dorsal interdigitating synchondroses begin to fail [33]. This results in loss of joint integrity and, consequently, treatment failure, which was observed in all specimens. Expressing rotational movement as angular displacement (degrees), rather than linear displacement (millimeters), is appropriate for rotational kinematics and allows for direct comparison between specimens with different sizes.

Yield force was calculated as:

F_t_ = F × (l_screw_/l_average_)

where F is the load (N) at yield, l_screw_ the individual moment arm (cm), and l_avergae_ the mean moment arm (cm).

Ultimate load was calculated as:

F_m_ = (M/l_average_)/cos (5°)

where M is torque force (N) at 5° displacement.

Stiffness was defined as the slope of the linear portion of the load–displacement curve up to the yield load.

Failure mode was recorded for each specimen.

### 2.7. Statistical Analysis

Data were analyzed using commercial statistical software (R version 4.5.0, (11 April 2025)). Body weight, moment arm, yield load, ultimate load, and stiffness were compared among the three groups. Normality was assessed using the Shapiro–Wilk test. Body weight, torque, yield load, and ultimate load were normally distributed. Homogeneity of variance was evaluated using Levene’s test. When equal variances were confirmed, a Fischer ANOVA was performed. Moment arm and stiffness were not normally distributed and were therefore analyzed using the Kruskal–Wallis test. Post hoc comparisons were performed using pairwise Dunn tests for non-parametric data and pairwise Student’s *t*-tests for Fischer ANOVA. Statistical significance was defined as *p* ≤ 0.05; 0.05 < *p* ≤ 0.10 was considered a statistical trend. Due to the small sample size, *p*-values were not corrected for multiple comparisons in order to maintain statistical power and to reduce the risk of Type 2 error.

## 3. Results

For insertion torque assessment, pelves from 10 cadaveric DSH cats were used (3 females, 7 males; age unknown; median body weight 4.2 kg, range 2.3–6.2 kg). For mechanical testing, 36 DSH cadavers were included (19 males, 17 females). Mean age was 13.3 years (range 3–20 years), but age was unknown in 12 cats and was therefore excluded from statistical analysis. Mean body weight was 3.7 kg (range 2.3–7.2 kg), with no significant difference among the groups (*p* = 0.73, effect size = 0.00). Screw lengths ranged from 20 to 26 mm across all groups (CCHS, PS, LS).

Median moment arm values were: 30.25 cm (IQR 3.3) for CCHS, 30.10 cm (IQR 3.4) for PS, and 31.40 cm (IQR 4.7) for LS. No significant difference was detected among groups (*p* = 0.19, effect size = 0.09).

**Figure 2:**
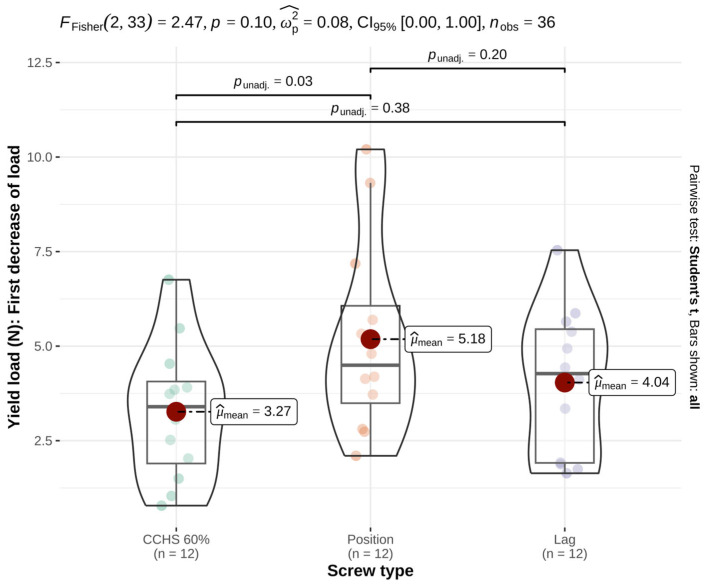
At the yield load, comparison of cannulated headless compression screws (CCHSs), cortical positional screws (PSs), and lag screws in a feline cadaveric SIL model showed no significant differences among groups, although a trend was observed (*p* = 0.10). Pairwise comparison revealed a significant difference between CCHS and PS (*p* = 0.03). Bars show mean values; significance defined as *p* ≤ 0.05; 0.05 < *p* ≤ 0.10 was considered a statistical trend.

At the yield load, mean values did not differ significantly among groups, although a trend was observed (*p* = 0.10, effect size = 0.08) (Table 1 and Figure 2). Pairwise comparison identified a significant difference between CCHS and PS (*p* = 0.03).

**Figure 3:**
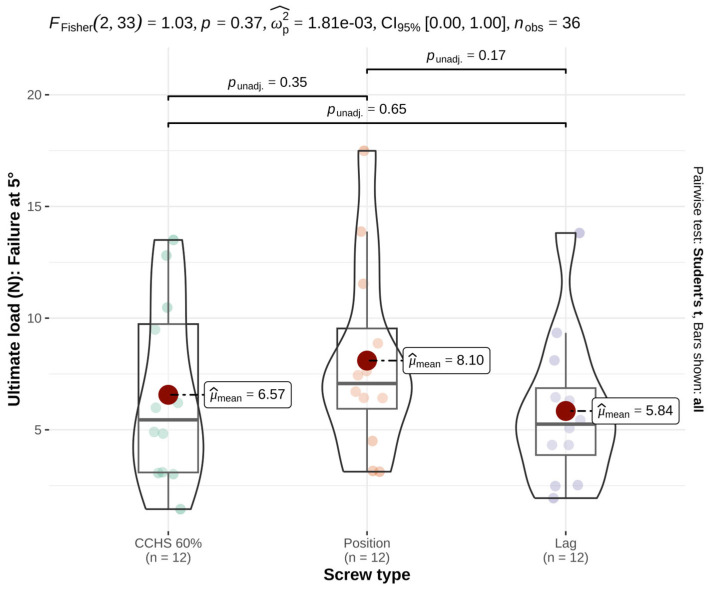
At the ultimate load, comparison of cannulated headless compression screws (CCHSs), cortical positional screws (PSs), and lag screws in a feline cadaveric SIL model showed no significant differences among groups (*p* = 0.37). Bars indicate mean values; significance defined as *p* ≤ 0.05.

At the ultimate load, the mean forces did not differ significantly among groups (*p* = 0.37, effect size = 0.00) (Table 1 and Figure 3).

**Figure 4:**
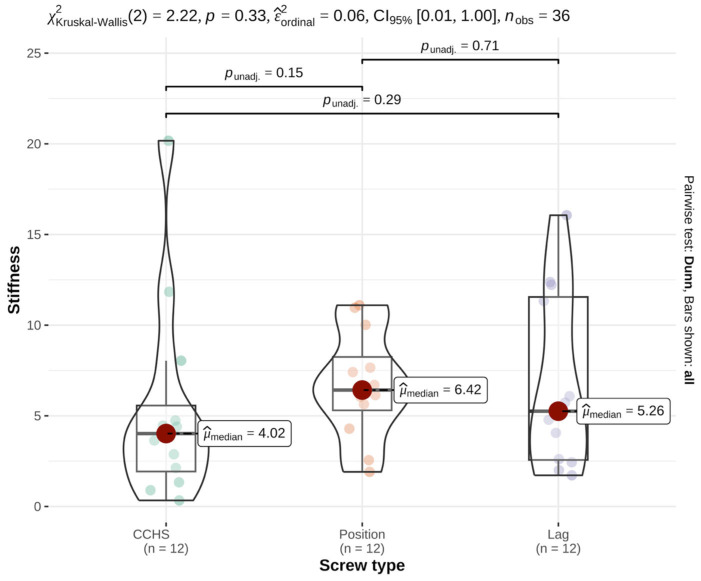
For rotational stiffness, comparison of cannulated headless compression screws (CCHSs), cortical positional screws (PSs), and lag screws in a feline cadaveric SIL model showed no significant differences among groups (*p* = 0.33). Bars show median values; significance defined as *p* ≤ 0.05.

Median stiffness values also did not differ significantly among groups (*p* = 0.33, effect size = 0.06) (Table 1 and Figure 4).

Failure analysis showed that all constructs failed by screw loosening, with no bone failure observed. No screws bent or fractured during testing. In the CCHS group, failure occurred due to rotation of the implant within the sacrum, while the threaded screw head remained anchored in the ilium. In contrast, failure in the PS and LS groups resulted from rotation of the ilium around the screws.

## 4. Discussion

The three screw types yielded comparable rotational strength in this cadaveric feline SIL model, with no statistically significant differences detected between groups. This outcome contradicted both hypotheses, namely that CCHSs would provide greater rotational stability than cortical screws and that lag screws would result in stronger fixation than positional screws.

This study provides the first evidence that cannulated partially threaded compression headless screws (CCHSs) and cortical screws offer comparable rotational stability in a feline SIL model. This finding was unexpected, as headless compression screws are designed to enhance interfragmentary compression, which is generally thought to increase fixation stability [17,18,19,20,21]. The results of the present study may be explained by the design of the CCHS, which is partially threaded. The threaded tip engages only cancellous bone within the central body of the sacrum, whereas the fully threaded cortical screw engages cancellous bone along its entire length, including the abaxial cortex of the sacrum. The lower cumulative bone–screw interface of the CCHS may therefore account for the comparable rotational resistance observed in this study. This interpretation is supported by the observed failure patterns. In the CCHS group, rotational failure occurred in the sacrum, the weaker component of the construct, while the threaded head remained securely anchored to the ilium. In contrast, the cortical screws, with more threads engaging additional sacral bone, the stronger part of the construct, demonstrated failure through rotation of the ilium around the unthreaded screw heads. These failure patterns align with those reported in a canine biomechanical study [22], supporting our interpretation. Based on this reasoning, a fully threaded cannulated HCS design may potentially improve the biomechanical effectiveness of HCS fixation in feline SIL. However, biomechanical studies evaluating fully threaded HCSs in feline SILs are not currently available. As minimally invasive HCS fixation becomes increasingly common, further biomechanical investigations of fully threaded cannulated HCSs are warranted to determine the optimal treatment strategy for feline SIL.

This study questions the assumption that a cortical lag screw provides superior stability in feline SIL. Notably, no significant difference was observed between cortical screws applied in either positional or lag fashion in this cadaveric feline SIL model. This finding may be explained by the additional engagement of the ilial cortices achieved by the positional screw. In contrast, lag screws use a glide hole to generate interfragmentary compression. Supporting this hypothesis, a biomechanical study in small dogs demonstrated that compression across the sacroiliac joint generated by lag screws did not improve construct stability or stiffness relative to cortical positional screws [23]. The authors proposed that the greater screw thread purchase in the ilium increased the stripping torque of the positional screws, thereby compensating for the higher frictional interface between the sacrum and the ilium produced by lag screws [23]. In general, sacroiliac lag screw fixation is intended to provide interfragmentary compression, thereby reducing micromotion and improving rotational resistance during weight bearing. Although the compressive performance of the screws tested in the present study has been challenged in a recent biomechanical study of feline SILs [30], these effects may not be detected in a single load-to-failure test. A human cadaveric study evaluated the compression performance of different lag screws across the sacroiliac joint over time and found a substantial decrease in compression, suggesting limited long-term compression [34]. Overall, further biomechanical studies comparing screws placed in lag and positional fashion under cyclic loading are therefore needed to better assess their performance under clinically relevant conditions.

A single-screw model was used in this study. Previous canine biomechanical research showed that increasing cortical screw size improves construct strength in bending and shear, but not in torsion [24]. In dogs, two-screw fixation provides greater rotational stability than a single larger screw [23,24]. In cats, placement of a second cortical screw is feasible, mainly using small-diameter screws [35]. However, failure of caudal screws has been reported in 4.7%, likely due to increased mechanical stress [35]. In cats, the safe anatomical corridor within the sacral body is narrow and becomes even more restricted caudoventrally [5], making placement of a second screw technically challenging. This limitation increases the risk of malposition, neural or vascular injury, and screw loosening [24,35]. In small dogs (approximately 6 kg), constructs using two 2.3 mm titanium HCS were 3.4 times stronger than a single 3.5 mm stainless-steel cortical lag screw [22]. Furthermore, fluoroscopic guidance for inserting cannulated headless compression screws (HCSs) over an accurately placed guide wire improves implant positioning in cats [14,15,16], and enables precise planning to avoid the ventral sacral foramina in small dogs, further supporting a minimally invasive approach [22,23]. These findings indicate the need for further biomechanical studies in cats to evaluate CCHSs of different numbers, sizes, and lengths to determine the optimal fixation strategy for feline SIL.

This study has several limitations. Rotational forces may biomechanically simulate weight bearing and are therefore likely among the most relevant forces acting on sacroiliac luxation stabilization [22,23,26]. However, the sacroiliac joint is exposed to complex multiplanar forces, including shear, bending, and rotation [24]. In addition, static load-to-failure testing does not reflect clinical failure mechanisms, such as implant stability and screw loosening. Furthermore, the experimental model used in the present study does not replicate a complete pelvic ring injury. Overall, additional biomechanical investigations—particularly those assessing dynamic contact [36] and cyclic loading in models that more closely replicate pelvic ring injuries—are needed and may better reflect clinically relevant screw performance.

Although cadaveric bone more closely approximates in vivo conditions than synthetic materials, heterogeneity remains a limitation in biomechanical studies using natural specimens. The variability observed in this study may be explained by differences in bone quality among specimens. The mean donor age and repeated freeze–thaw cycles may also have influenced the mechanical performance and contributed to data variability. However, feline cadavers are typically obtained from older donors, and previous work suggests that multiple freeze–thaw cycles do not significantly affect biomechanical properties [37]. Pre-testing assessment of bone density and group stratification [38,39] may therefore reduce donor-related heterogeneity and should be considered in future studies to strengthen validity.

The free-hand drilling technique may have introduced variability in screw trajectory and depth, potentially affecting the biomechanical results. Drill guides could allow for more standardized screw insertion [23]. However, individual differences in sacral morphology and optimal entry points were observed in some specimens, consistent with previous reports [40]. The use of standardized drill guides might therefore have resulted in incorrect placement in some cases and influenced the results. Anatomical variability has also been reported in a canine biomechanical study, where it led to modifications of the luxation model [25]. In that study, holes were drilled under fluoroscopic guidance before creating the luxation model, which may have improved consistency [25]. In the present study, individualized drilling and fluoroscopic verification prior to creating the luxation model likely contributed to increased precision.

This investigation was designed as a pilot study because no prior data were available for power calculation. Sample size was based on published recommendations [41,42]. A trend was observed for yield load among groups (*p* = 0.10), with a significant difference identified in pairwise comparison. The latter finding may represent a type I error. The decision not to apply a correction for multiple comparisons to reduce the risk of type II error may have increased the susceptibility to type I error. However, achieving an optimal balance between these errors through an adequate sample size was not feasible due to budget constraints, as increasing the number of specimens would have made the study unaffordable. Overall, statistically significant differences among groups in yield and ultimate load may not have been detected due to the limited sample size. Increasing the sample size would improve statistical power and may help to clarify differences between CCHSs and cortical positional screws, providing more clinically relevant results.

Another limitation is the presence of a pressure-mapping sensor, as compressive forces were measured in the same specimens prior to mechanical testing. Screw extraction and reinsertion to remove the sensor were avoided because this would likely have altered local tribological conditions at the screw–bone interface, impairing stability and compromising comparability. However, combining compressive force measurements with mechanical testing is common in biomechanical research and has been applied in human cadaveric studies assessing stability and dynamic contact loading of different surgical techniques [36]. Furthermore, initial compressive force measurements did not compromise specimen integrity for subsequent mechanical testing. As this limitation affected all specimens equally, valid comparisons between groups remained possible.

## 5. Conclusions

Cannulated compression headless screws, cortical positional screws, and cortical lag screws showed comparable rotational strength and stiffness under the single load-to-failure conditions used in this cadaveric simulated feline SIL model. From a biomechanical perspective, these findings question the assumed advantage of compression screws and suggest that fully threaded positional screws may represent a valid option in feline SIL.

However, further biomechanical studies evaluating compressive forces under dynamic, simulated weight-bearing conditions, as well as studies with larger sample sizes, are needed to assess a potential advantage of fully threaded positional screws and to more comprehensively evaluate their performance in the minimally invasive treatment of unilateral feline SILF.

## Figures and Tables

**Table 1:** Yield load, ultimate load, and rotational stiffness: comparison of cannulated compression headless screws (CCHSs), cortical positional screws, and cortical lag screws in a feline cadaveric model of SIL (*n* = 12 per group). The table represents the individual data and the summarized results in bold, reported as mean ± SEM and median (IQR).

Screw Type	Yield Load	Ultimate Load	Stiffness
CCHS 1	3.74	106.45	4.46
CCHS 2	0.78	55.31	1.33
CCHS 3	4.54	371.64	11.84
CCHS 4	2.03	89.00	4.40
CCHS 5	1.04	42.06	0.34
CCHS 6	2.52	89.97	0.90
CCHS 7	6.76	391.83	20.17
CCHS 8	3.85	140.20	4.74
CCHS 9	3.91	304.03	3.64
CCHS 10	5.47	180.13	8.04
CCHS 11	3.06	173.84	2.88
CCHS 12	1.50	275.39	2.13
**Mean/Median**	**3.27 ± 1.7**	**6.57 ± 3.9**	**4.02 (3.6)**
Positional screw 1	9.32	13.88	7.66
Positional screw 2	2.10	8.87	2.55
Positional screw 3	3.72	6.42	1.91
Positional screw 4	10.20	11.54	11.10
Positional screw 5	2.81	3.16	10.96
Positional screw 6	4.19	7.44	6.70
Positional screw 7	4.80	7.63	10.02
Positional screw 8	2.74	3.13	7.41
Positional screw 9	7.18	17.49	6.05
Positional screw 10	4.14	4.50	4.29
Positional screw 11	5.70	6.71	6.14
Positional screw 12	5.33	6.43	5.64
**Mean/Median**	**5.18 ± 2.5**	**8.10 ± 4.1**	**6.42 (2.9)**
Lag screw 1	5.65	8.11	12.38
Lag screw 2	7.54	9.34	16.06
Lag screw 3	1.92	5.07	2.61
Lag screw 4	4.94	6.31	11.33
Lag screw 5	5.87	13.81	12.22
Lag screw 6	5.38	6.46	5.73
Lag screw 7	1.75	1.94	4.78
Lag screw 8	3.35	4.32	2.44
Lag screw 9	4.43	4.32	6.08
Lag screw 10	1.88	2.52	2.00
Lag screw 11	1.64	2.48	1.72
Lag screw 12	4.11	5.44	4.06
**Median/Median**	**4.04 ± 1.9**	**5.84 ± 3.2**	**5.26 (9.0)**
***p*** **-value**	**0.10**	**0.37**	**0.33**

## Data Availability

Additional research data, including analytic methods, raw data, processed data, and study material, is available upon request to interested researchers.

## Abbreviations

The following abbreviations are used in this manuscript:

SILF	Sacroiliac luxation fracture
HCS	Headless compression screw
CCHS	Cannulated compression headless screw
PS	Positional screw
LS	Lag screw
IQR	Interquartile range

## References

[1] Bookbinder P.F., Flanders J.A. (1992). Characteristics of pelvic fracture in the cat. Vet. Comp. Orthop. Traumatol..

[2] Meeson R.L., Geddes A.T. (2017). Management and long-term outcome of pelvic fractures: A retrospective study of 43 cats. J. Feline Med. Surg..

[3] Bird F.G., de Vicente F. (2020). Conservative management of sacroiliac luxation fracture in cats: Medium- to long-term functional outcome. J. Feline Med. Surg..

[4] Meeson R., Corr S. (2011). Management of pelvic trauma. J. Feline Med. Surg..

[5] Burger M., Forterre F., Brunnberg L. (2004). Surgical anatomy of the feline sacroiliac joint for lag screw fixation of sacroiliac fracture-luxation. Vet. Comp. Orthop. Traumatol..

[6] Langley-Hobbs S.J. (2010). Sacroiliac luxation in cats. Proceedings of the ACVS Veterinary Symposium, Seattle, WA, USA, 18–23 October 2010.

[7] Montavon P.M., Messmer M. (2004). Pelvic fractures in the dog and cat: A classification system and review of 556 cases. Vet. Comp. Orthop. Traumatol..

[8] Yap F.W., Dunn A.L., Farrell M., Calvo I. (2014). Trans-iliac pin/bolt/screw internal fixation for sacroiliac luxation or separation in cats: Six cases. J. Feline Med. Surg..

[9] Jacobson A., Schrader S.C. (1987). Peripheral nerve injury associated with fracture or fracture-dislocation of the pelvis in dogs and cats: 34 cases (1978–1982). J. Am. Vet. Med. Assoc..

[10] Tomlinson J. (2012). Minimally invasive repair of sacroiliac luxation in small animals. Vet. Clin. N. Am. Small Anim. Pract..

[11] Raffan P.J., Joly C.L., Timm P.G., Miles J.E. (2002). A tension band technique for stabilisation of sacroiliac separations in cats. J. Small Anim. Pract..

[12] Moens N.M.M., DeCamp C.E., Johnston S.A., Tobias K.M. (2018). Fractures of the Pelvis. Veterinary Surgery: Small Animal.

[13] Shales C., Moores A., Kulendra E., White C., Toscano M., Langley-Hobbs S. (2010). Stabilization of sacroiliac luxation in 40 cats using screws inserted in lag fashion. Vet. Surg..

[14] Rollins A., Balfour R., Szabo D., Chesvick C.M. (2019). Evaluation of fluoroscopic-guided closed reduction versus open reduction of sacroiliac fracture-luxations stabilized with a lag screw. Vet. Comp. Orthop. Traumatol..

[15] Jourdain M., Fernandes D., Védrine B., Gauthier O. (2024). Fluoroscopically-assisted closed reduction and percutaneous fixation of sacroiliac luxations in cats using 2.4 mm headless cannulated compression screws: Description, evaluation and clinical outcome. Vet. Surg..

[16] de Jong L., Proot J.L.J., Pillin L.J.P., Janssens L.A.A. (2025). Minimally Invasive Placement of Cannulated Headless Compression Screws for Reduction of Sacroiliac Luxation in 14 Cats. Vet. Comp. Orthop. Traumatol..

[17] Fowler J.R., Ilyas A.M. (2010). Headless Compression Screw Fixation of Scaphoid Fractures. Hand Clin..

[18] Adla D.N., Kitsis C., Miles A.W. (2005). Compression forces generated by Mini bone screws—A comparative study done on bone model. Injury.

[19] Assari S., Darvish K., Ilyas A.M. (2012). Biomechanical analysis of second-generation headless compression screws. Injury.

[20] Gruszka D., Burkhart K., Nowak T., Achenbach T., Rommens P., Müller L. (2012). The Durability of the Intrascaphoid Compression of Headless Compression Screws: In Vitro study. J. Hand Surg..

[21] Hart M.B., Wu J.J., Chao E.Y., Kelly P.J. (1985). External skeletal fixation of canine tibial osteotomies. Compression compared with no compression. J. Bone Jt. Surg..

[22] Kang A., Lee H., Lee A., Roh Y., Sim B., Jeong J. (2024). Biomechanical Comparison of Double 2.3-mm Headless Cannulated Self-Compression Screws and Single 3.5-mm Cortical Screw in Lag Fashion in a Canine Sacroiliac Luxation Model: A Small Dog Cadaveric Study. Vet. Comp. Orthop. Traumatol..

[23] Hanlon J., Hudson C.C., Litsky A.S., Jones S.C. (2022). Mechanical evaluation of canine sacroiliac joint stabilization using two short screws. Vet. Surg..

[24] Radasch R.M., Merkley D.F., Hoefle W.D., Peterson J. (1990). Static strength evaluation of sacroiliac fracture-separation repairs. Vet. Surg..

[25] Bae S., Jeon Y., Lee H., Jeong J. (2025). Effect of thread direction on rotational stability in lag-screw fixation of sacroiliac luxation: An ex vivo cadaveric study in small-breed dogs. Vet. Surg..

[26] Jang S.-K., Lee Y., Lee S., Do J., Kim J.-W., Cho J.-S., Kim H.-Y., Kim J.-M. (2026). Double J-shaped Kirschner wire fixation provides superior biomechanical stability for feline sacroiliac luxation compared with conventional techniques. Am. J. Vet. Res..

[27] Depuy Synthes (2019). Cannulated Compression Headless Screws (CCHS). 2.0, 2.5, 3.0, 3.5, 4.0, 4.5, 5.5, 6.5, 7.5 mm. Surgical Technique. https://a490ebc3686a416702a4-875c9cad01df4876deaf64b6bbcf2205.ssl.cf2.rackcdn.com/ota_3faeb208bd6827b31b625d3a475a1d20.pdf.

[28] Fletcher J.W.A., Zderic I., Gueorguiev B., Richards R.G., Gill H.S., Whitehouse M.R., Preatoni E. (2020). Stripping torques in human bone can be reliably predicted prior to screw insertion with optimum tightness being found between 70% and 80% of the maximum. Bone Jt. Res..

[29] Macpherson J.M., Ye Y. (1998). The cat vertebral column: Stance configuration and range of motion. Exp. Brain Res..

[30] Wachsmuth L., Wieser J., Thorwächter C., Zablotski Y., Lorenz N.D., Lauer S., Kornmayer M. (2026). Interfragmentary compression in a feline sacroiliac luxation model: Biomechanical comparison of cannulated compression headless screws and cortical screws applied in positional or lag fashion. BMC Veter. Res..

[31] Sturesson B., Selvik G., Uden A. (1989). Movements of the sacroiliac joints. A roentgen stereophotogrammetric analysis. Spine.

[32] Shin S., Kwak D.-S., Lee U.-Y. (2024). Mobility and anthropometry of the sacroiliac joint: Range of motion and morphological characteristics. Biomed. Eng. Lett..

[33] Dennis R., Kirberger R.M., Barr F., Wrigley R.H., Dennis R., Kirberger R.M., Barr F., Wrigley R.H. (2010). Appendicular skeleton. Handbook of Small Animal Radiology and Ultrasound Techniques and Differential Diagnoses.

[34] Chatain G.P., Oldham A., Uribe J., Duhon B., Gardner M.J., Witt J.-P., Yerby S., Kelly B.P. (2023). Biomechanics of sacroiliac joint fixation using lag screws: A cadaveric study. J. Orthop. Surg. Res..

[35] Schreiber K.R.L., Thibault A., Hamon M., Haudiquet P. (2024). Stabilization of 82 sacroiliac luxations in 67 cats using two sacroiliac screws (2014-2023). Vet. Surg..

[36] Ritter D., Hachem A.-I., Scheibel M., Raiss P., Denard P.J., Campagnoli A., Wijdicks C.A., Bachmaier S. (2023). Primary Stability and Bone Contact Loading Evaluation of Suture and Screw based Coracoid Graft Fixation for Anterior Glenoid Bone Loss. Am. J. Sports Med..

[37] Kang Q., An Y.H., Friedman J. (1997). Effects of multiple freezing-thawing cycles on ultimate indentation load and stiffness of bovine cancellous bone. Am. J. Vet. Res..

[38] Müller J.-U., Nowak S., Matthes M., Pillich D.T., Schroeder H.W.S., Müller J. (2024). Biomechanical comparison of two different compression screws for the treatment of odontoid fractures in human dens axis specimen. Clin. Biomech..

[39] Pollard J.D., Deyhim A., Rigby R.B., Dau N., King C., Fallat L.M., Bir C. (2010). Comparison of pullout strength between 3.5-mm fully threaded, bicortical screws and 4.0-mm partially threaded, cancellous screws in the fixation of medial malleolar fractures. J. Foot Ankle Surg..

[40] Shales C.J., White L., Langley-Hobbs S.J. (2009). Sacroiliac Luxation in the Cat: Defining a Safe Corridor in the Dorsoventral Plane for Screw Insertion in Lag Fashion. Vet. Surg..

[41] Julious S.A. (2005). Sample size of 12 per group rule of thumb for a pilot study. Pharm. Stat..

[42] Hertzog M.A. (2008). Considerations in determining sample size for pilot studies. Res. Nurs. Health.

